# Resonant Tunnelling and Intersubband Optical Properties of ZnO/ZnMgO Semiconductor Heterostructures: Impact of Doping and Layer Structure Variation

**DOI:** 10.3390/ma17040927

**Published:** 2024-02-17

**Authors:** Aleksandar Atić, Xizhe Wang, Nikola Vuković, Novak Stanojević, Aleksandar Demić, Dragan Indjin, Jelena Radovanović

**Affiliations:** 1 School of Electrical Engineering, University of Belgrade, Bulevar Kralja Aleksandra 72, 11120 Belgrade, Serbia; atic@vin.bg.ac.rs (A.A.); nvukovic@etf.bg.ac.rs (N.V.); novak.stanojevic@vlatacom.com (N.S.); 2 Vinča Institute of Nuclear Sciences, National Institute of Republic of Serbia, University of Belgrade, Mike Petrovića Alasa 12-14, Vinča, 11351 Belgrade, Serbia; 3 Centre for Light-Based Research and Technologies Coherence, Mike Petrovića Alasa 12-14, 11351 Belgrade, Serbia; 4 School of Electronic and Electrical Engineering, University of Leeds, Woodhouse Lane, Leeds LS2 9JT, UK; el17xw@leeds.ac.uk (X.W.); a.demic@leeds.ac.uk (A.D.); d.indjin@leeds.ac.uk (D.I.); 5 Vlatacom Institute of High Technologies, Bulevar Milutina Milankovića 5, 11070 Belgrade, Serbia

**Keywords:** wide-bandgap oxide semiconductors, resonant tunnelling, intersubband transitions, depolarisation shift

## Abstract

ZnO-based heterostructures are up-and-coming candidates for terahertz (THz) optoelectronic devices, largely owing to their innate material attributes. The significant ZnO LO-phonon energy plays a pivotal role in mitigating thermally induced LO-phonon scattering, potentially significantly elevating the temperature performance of quantum cascade lasers (QCLs). In this work, we calculate the electronic structure and absorption of ZnO/ZnMgO multiple semiconductor quantum wells (MQWs) and the current density–voltage characteristics of nonpolar m-plane ZnO/ZnMgO double-barrier resonant tunnelling diodes (RTDs). Both MQWs and RTDs are considered here as two building blocks of a QCL. We show how the doping, Mg percentage and layer thickness affect the absorption of MQWs at room temperature. We confirm that in the high doping concentrations regime, a full quantum treatment that includes the depolarisation shift effect must be considered, as it shifts mid-infrared absorption peak energy for several tens of meV. Furthermore, we also focus on the performance of RTDs for various parameter changes and conclude that, to maximise the peak-to-valley ratio (PVR), the optimal doping density of the analysed ZnO/Zn_88_Mg_12_O double-barrier RTD should be approximately $10^{18}\;{cm}^{-3}$, whilst the optimal barrier thickness should be 1.3 nm, with a Mg mole fraction of ~9%.

## 1. Introduction

The demand for materials tailored to the mid-infrared (MIR) and terahertz (THz) spectral ranges is on the rise, leading to a growing number of applications [1,2,3,4,5]. Within these spectrum ranges, semiconductor materials [6,7,8], especially semiconductor heterostructures and superlattices (SLs), present an intriguing avenue for exploring and regulating carrier quantum transport and optical transitions in both radiation sources and detectors [9,10,11,12,13,14,15,16,17]. In materials science, an SL typically denotes a periodic arrangement of alternating materials. Following the recent progress in the near-infrared spectral range, semiconductor SL structures hold promise for extending innovative capabilities into the MIR and THz domains [18,19,20,21,22,23]. Additionally, modern epitaxial growth techniques used to create quantum cascade lasers establish a highly competitive technology for the MIR and THz ranges [24,25,26].

Furthermore, linear and nonlinear optical properties in quantum heterostructures, like SLs and quantum wells based on wide-bandgap oxide semiconductors, are the focus of research due to their potential applications in optoelectronics, such as QCLs [1,27,28,29] and RTDs [30]. The properties of these devices are based on two quantum phenomena: electronic confinement and tunnelling. Intersubband transitions (ISBTs) are typically collective effects that involve large electron densities of interacting particles, and the most important manifestation of this collective character is that, in the presence of electromagnetic radiation, each electron is affected by an effective field induced by the excitation of the other electrons, called a depolarisation field [31,32].

GaAs-based QCLs are the most promising devices emitting in the terahertz frequency range, but they have lacked significant improvements in recent years and are still limited to operating at low temperatures (~260 K) [33]. They are fundamentally limited by electron-longitudinal-optical (LO) phonon resonance at around 36 meV in GaAs, causing parasitic nonradiative depopulation of the upper laser level at room temperature. The 260 K record performance has been established due to a paradigm shift in designing structures beyond LO-phonon resonance energy [34,35], however, the fundamental limit lies in nonradiative electron–LO-phonon scattering between the lasing levels [35], and this can only be mitigated by using material systems with larger resonant LO-phonon energy. Promising alternative semiconductors to solve this problem include new material systems like zinc oxides (ZnO) with their larger LO-phonon energy (~72 meV) [27]. ZnO with a hexagonal wurtzite structure is currently emerging as a promising II–VI direct wide-bandgap semiconductor for its use in photonic devices, such as LEDs, solar cells, thin film transistors and other heterostructures [36,37,38,39]. High resonant electron–LO-phonon energy in ZnO-based compounds is just one important beneficial property, and their large bandgap, high conduction band offset and resistance to electric breakdown are other relevant benefits [39,40,41,42,43,44,45]. Furthermore, prospective ZnO-based lasers can cover a 5–12 THz emission frequency range [46], an important range relevant for the detection and imaging of explosives, which cannot be covered by standard GaAs-based THz QCLs. Recently achieved progress in the growth of low-density defect nonpolar m-plane ZnO-based heterostructures [47] opens a perspective towards the demonstration of ZnO-based unipolar structures capable of operating at an elevated or even room temperature.

Sizeable optical phonon energy in ZnO-based structures should facilitate the population inversion for ISBTs with energy well below the optical phonon energy [47]. Despite significant advances in the reproducibility and the stability of the p-doping of ZnO, it remains a considerable challenge, which strongly limits the development of this wide-bandgap oxide semiconductor for bipolar electrical devices [47]. Still, it may be possible to use ZnO-based heterostructures for unipolar devices (with only n-type doping), such as RTDs, quantum-well infrared photodetectors and quantum cascade detectors or lasers [47]. To master the fabrication of ZnO-based quantum cascade structures, a high-quality epitaxial growth is crucial, combined with a well-controlled fabrication process, including (selective) Zn(Mg)O etching and the deposition of low-resistance ohmic contacts. V. Sirkeli et al. reported a numerical study of the negative differential resistance in nonpolar m-plane ZnO/ZnMgO THz RTDs with double- and triple-quantum barriers [48]. They showed that by optimising the design structure of RTDs, the constituent layer material, its width and the doping level, the mW level of the output power of terahertz emissions from these devices can be achieved at room temperature [48]. Liu et al. investigated THz intersubband absorption in step quantum well structures based on ZnO/ZnMgO materials at 77 K [49]. Recently, Meng et al. demonstrated the first intersubband electroluminescence from nonpolar m-plane ZnO QC structures [46].

In this paper, we numerically investigate the different combinations of ZnO/ZnMgO multi-quantum-well and resonant-tunnelling structures to analyse the sensitivity of the position and magnitude of the intersubband absorption peak and the tunnelling current peak-to-valley ratio on the monolayer-scale layer structure, Mg composition fluctuation and doping density variation.

## 2. Methods

We start from the one-dimensional envelope function effective-mass Schrödinger equation:

(1) $$-\frac{\hbar^{2}}{2}\frac{d}{dz}\frac{1}{m^{*}(z)}\frac{d\psi_{i}(z)}{dz}+U_{eff}\left(z\right)\psi_{i}\left(z\right)=E\psi_{i}\left(z\right),\;$$

where $\psi_{i}(z)\;$ is the envelope wave function; *E* is the eigenvalue of the electron energy; $m^{*}$ is the electron’s effective mass; and ${\;U}_{eff}\left(z\right)$ is the total effective potential energy, which is given as the following:

(2) $$U_{eff}\left(z\right)=U_{c}\left(z\right)-e\varphi \left(z\right)+U_{xc}\left(z\right)-eFz\;$$

where $U_{c}$ is the conduction band edge of the heterostructure, *F* is the externally applied electric field and $\varphi \left(z\right)$ is the electrostatic potential. $U_{xc}$ is the local exchange-correlation potential, as described in Appendix A. The ZnO band structure indicates very high neighbouring valleys, as illustrated, for example, in [50], where the higher valley minima would be ~1.5–2 eV above the G valley; thus, we expect no or very small band-mixing effects. Therefore, the use of a single-band envelope function effective-mass model here is justified.

The total effective potential energy depends on the envelope functions in a semiconductor heterostructure, and the system of Schrödinger–Poisson equations needs to be solved self-consistently. The electrostatic potential of the Poisson equation reads as follows:

(3) $$\frac{d^{2}\varphi (z)}{dz^{2}}=\frac{e}{\varepsilon (z)}\left(n\left(z\right)-N_{D}\left(z\right)\right),\;$$

where, as above, φ(z) is the electrostatic potential, ε(z) is the dielectric constant and $N_{D}\left(z\right)$ is the doping concentration. In a semiconductor MQW-based heterostructure bound electron energies can, therefore, be calculated fully quantum mechanically, and the electron density $n\left(z\right)$ is given as follows:

(4) $$n\left(z\right)=\sum_{i}N_{s,i}{\left|\psi_{i}\left(z\right)\right|}^{2},\;$$

where $N_{s,\;i}$ is the sheet carrier density corresponding to the i-th electron bound state, which is defined as follows:

(5) $$N_{s,i}=\frac{m_{ti}k_{B}T}{\pi \hbar^{2}}\ln\left(1+e^{\frac{E_{F}-E_{i}\left(0\right)}{k_{B}T}}\right).\;$$

In the above equation, $E_{F}$ is the Fermi energy, $E_{i}(0)$ is the quasi-bound state energy for the zero transversal wave vector $\left(k_{t}=0\right),\;\;k_{B}$ is the Boltzmann constant and *T* is the crystal lattice’s absolute temperature. $m_{ti}$ is the transversal mass, defined as follows:

(6) $$\frac{1}{m_{ti}}=\int \psi_{i}^{*}(k_{t}=0)\frac{1}{m^{*}(z)}\psi_{i}(k_{t}=0)dz.\;$$

In finite gap semiconductors, nonparabolicity is typically characterised by the energy-dependent effective mass [51,52]. It is taken as a weak effect here, as it must be sufficiently close to the band edge with a finite gap. The material system that we consider in this work has a wide energy gap; thus, the band nonparabolicity can be neglected.

In quantum heterostructures based on potential barriers like resonant tunnelling structures, all electron energy levels belong to a continual spectrum, and the resonant electron states can be quantified by the tunnelling coefficient, τ(E). If the electric field (i.e., terminal voltage) *F* is applied across the structure, the current density can be calculated using the Esaki–Tsu formula [53]. This simplified approach assumes a coherent picture of electron tunnelling, using the approximation that electron transport is not affected by any phase-coherence breaking scattering effects [54]. This carrier transport model has been commonly used to characterise resonant tunnelling structures based on different material systems [54,55,56]. On the basis of these assumptions, the current density in the tunnelling structure can be calculated as follows:

(7) $$J=\frac{ek_{B}T}{2\pi^{2}\hbar^{3}}\int_{E_{c}}^{\infty }m^{*}\tau \left(E\right)ln\left[\frac{1+e^{\frac{E_{F}-E}{k_{B}T}}}{1+e^{\frac{E_{F}-E-eV_{R}}{k_{B}T}}}\right]dE$$

where for the reference level $E_{c}=0$, the conduction band minima can be used; τ(E) is the transmission (i.e., tunnelling) coefficient;$\;E_{F}$ is the Fermi energy in the highly doped emitter of resonant tunnelling structure; $V_{R}$ is the potential drop across the structure (such that $V_{R}=F\times lenght\;of\;resonant\;structure$); and $m^{*}$ is the effective mass in the well material. The Fermi energy, $E_{F}$, is calculated here using Fermi–Dirac statistics in a highly doped emitter/collector, assuming that all donors are ionised, i.e., the electron concentration in the emitter/collector is $n=N_{D}$ [57]. The magnitude of the current density (obtained from this coherent electron transport model) does not take into account other contributing factors to the total current, such as the scattering current and the thermionic current; thus, only the relative trends in the carrier transport and the possible negative differential resistivity behaviour can be identified and predicted in a prospective experiment. We can also express the electron density in the resonant tunnelling structure as follows [54,56]:

(8) $$n\left(z\right)=\frac{k_{B}T}{2^{\frac{3}{2}}\pi^{2}\hbar^{3}}\int_{E_{c}}^{\infty }{\left|\psi \left(z,E\right)\right|}^{2}{{(m}^{*})}^{\frac{3}{2}}E^{-\frac{1}{2}}\ln\left[1+e^{\frac{\left(E_{F}-E\right)}{k_{B}T}}\right]dE+\;\frac{k_{B}T}{2^{\frac{3}{2}}\pi^{2}\hbar^{3}}\int_{E_{c}-eV_{R}}^{\infty }{\left|\psi \left(z,E\right)\right|}^{2}{{(m}^{*})}^{\frac{3}{2}}E^{-\frac{1}{2}}ln[1+e^{\frac{\left(E_{F}-E-eV_{R}\right)}{k_{B}T}}]dE\;$$

In a multi-quantum-well structure, the subbarrier energy spectra, in a good approximation, can be assumed as discrete. The energies and wave functions of the bound states found from the Schrödinger–Poisson solver are further used to calculate the optical absorption, A(ωℏ), for the intersubband transitions. In the single-particle picture, the absorption coefficient is [32] calculated as follows:

(9) $$\alpha_{2D,s}(\omega )=C_{s}\sum_{\alpha }f_{\alpha }∆N_{\alpha }L(\omega -\omega_{\alpha }),\;$$

where $C_{s}$ is a constant, *f_α_* is the oscillator strength of the transition α and $L(\omega -\omega_{\alpha })$ is a Lorentzian centred in the intersubband transition frequency, $\omega_{\alpha }$.

In a situation in which a single subband is occupied, a blue shift of the absorption peak is observed (relative to the transition frequency), corresponding to the excitation of a collective mode of the system, called the intersubband plasmon [32]. In the case that the confined levels in the QW are closely spaced together and more than one of them is populated by electrons, several intersubband transitions occur simultaneously, resulting in an optical spectrum that consists of a single resonance whose energy is entirely different in comparison with the bare intersubband transitions. The resonance corresponds to the excitation of a collective mode of the system, the multisubband plasmon, resulting from the phase locking of all different intersubband transitions. Multisubband plasmons have been the subject of intense research over the last decade [58] and have proven to be an excellent platform for investigating the ultrastrong coupling of light and matter excitations in an optical cavity. Multisubband plasmon can be imagined as a charge density wave where the collective dipole oscillates along the growth direction of the quantum well (*z*-axis), whilst the plasmon propagates in the quantum well plane (x-y plane), with a characteristic in-plane wavevector [59].

The absorption coefficient can be calculated by integrating all of the current densities associated with different multisubband plasmons (see Appendix B):

(10) $$\alpha_{2D,m}\left(\omega \right)=C_{m}\sum_{n}\frac{1}{W_{n}}{\left|\int_{-\infty }^{+\infty }J_{n}\left(z\right)dz\right|}^{2}L\left(\omega -W_{n}\right)=C_{m}\sum_{n}W_{n}F_{n}L\left(\omega -W_{n}\right),\;$$

where $C_{m}$ is a constant, $W_{n}F_{n}$ is the effective oscillator strength for the n-th multisubband plasmon mode and *L* is a Lorentzian (or Gaussian) centred at the multisubband plasmon frequency, $W_{n}.$ Each effective oscillator strength results from the contribution of all of the optically active intersubband plasmons. They are weighted by the different quantities associated with individual transitions, such as dipole matrix elements or transition frequencies. They also depend on the coupling among the intersubband plasmons, which enters through the eigenvectors of the matrix, *M*. The coupling among the intersubband plasmons results in a redistribution of the absorption amplitude from the intersubband transitions to the multisubband plasmon modes. The total absorption satisfies the conservation of the total transition probability:

(11) $$\sum_{\alpha }\omega_{\alpha }{\left|z_{\alpha }\right|}^{2}∆N_{\alpha }=\sum_{n}W_{n}F_{n}.\;$$

where $z_{\alpha }$ represents a dipole matrix element of the transition α.

## 3. Results and Discussion

### 3.1. Multiple-Quantum-Well Structure

In the first part of this section, we compare the results of the ISBT optical absorption simulation in the multiple-QW structure analogues to those of the structure introduced in [46], which could serve as one period in a QCL active region. The well material was ZnO, whilst the barrier material was Zn_88_Mg_12_O. The structure was grown on a nonpolar m-plane ZnO substrate. The appearance of cracks in the epitaxial layer grown on a mismatched substrate—such as (Zn, Mg)O on ZnO—can be predicted using the critical thickness criterion, as provided in Reference [60]. The critical thickness is defined as the maximum thickness that can be grown before the nucleation of the first crack in the layer. It turns out that for the THz cascade device samples (which have a low Mg content range), relaxation on the m-plane was not a problem, because the critical thickness for 15% of Mg is above 1 µm, which allows for the growth of THz cascade devices made from m-plane ZnO and (Zn, Mg)O without defects. On the other hand, the realisation of a QCL in the IR range using the m-plane is not possible, because the Mg content is higher, and, consequently, the critical thickness is greatly reduced.

**Figure 1:**
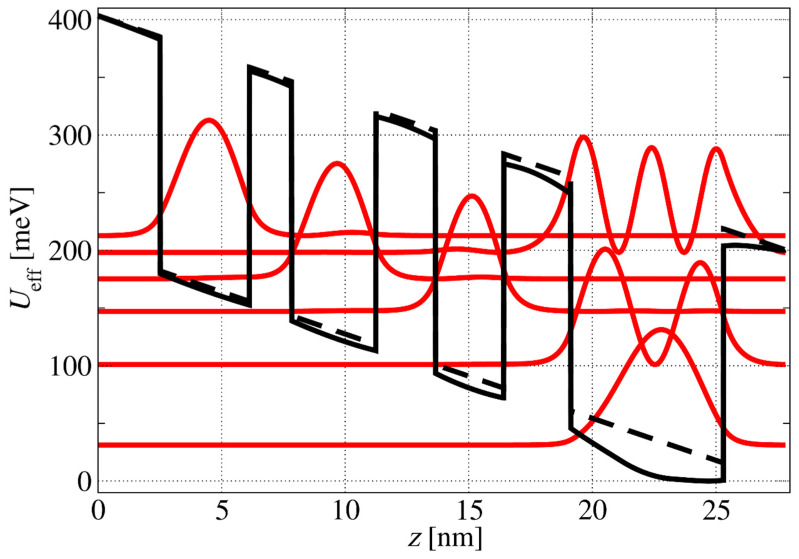
Conduction band diagram of a ZnO/Zn_88_Mg_12_O multiple-QW structure in an applied electric field. The layer sequence of the structure in nanometres, from left to right, is **2.5**/3.6/**1.7**/3.4/**2.4**/2.75/**2.7**/6.15/**2.5**, where the barriers are in bold, and the nonbold characters are ZnO wells. The centre (2.05 nm) of the 6.15 nm well (underlined) is doped with Ga to $N_{D}=3\times 10^{18}\;{cm}^{-3}.$ Bound states and their corresponding wave functions squared are denoted by solid red lines. The dashed black lines show the effective potential energy without the effect of well doping, while the full black lines refer to the case when doping effects are taken into consideration.

A temperature of T = 300 K and an operating external electric field of $F=73\;\frac{kV}{cm}$ were set for all simulations. Figure 1 shows the conduction band diagram of the structure. The effective masses in the well and barrier were taken as equal, reading as $m^{*}=0.28\;m_{0}$. The effective masses of the QWs and the barriers were taken to be the same as that of the ZnO polaron mass provided in [61] due to the strong interaction between electrons and phonons in this highly ionic material. The assumption of equal effective masses in the well and barrier did not introduce a significant error, since the Mg content was taken to be approximately 12%, leading to a conduction band offset of ~200 meV.

The conduction band offset is calculated as $∆E_{c}=0.675\;∆E_{g}$ [61,62], where $∆E_{g}$ is the difference in the band gap between the two semiconductors in the junction and is calculated as 25 meV multiplied by the % of the Mg in the barrier.

**Figure 2:**
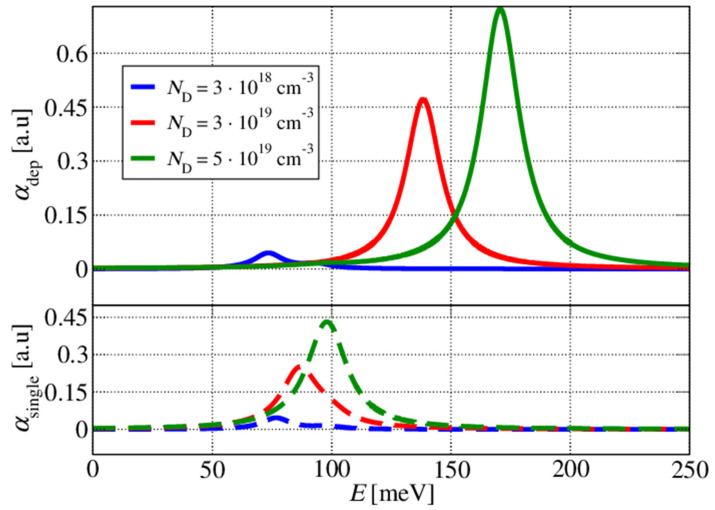
Intersubband absorption spectra for differing values of wide well doping in the structure shown in Figure 1. The upper diagram shows the absorption spectra with the depolarisation shift, whilst the lower one shows the absorption spectra calculated for a single-plasmon picture.

**Figure 3:**
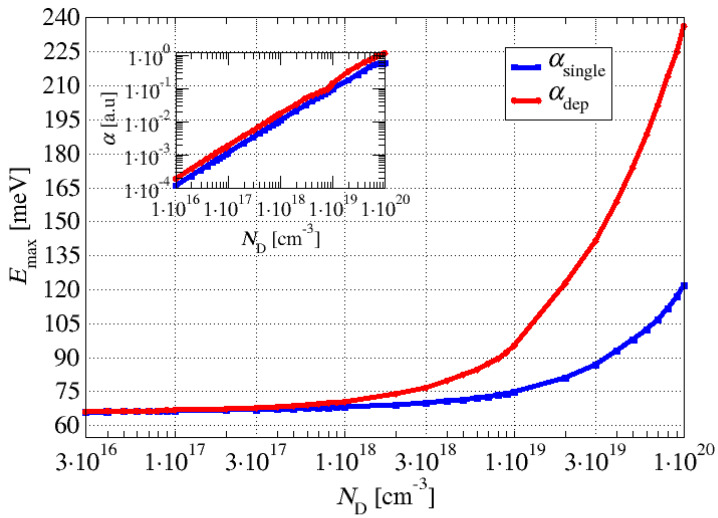
Absorption peak energy as a function of the wide well doping density in the structure shown in Figure 1. The blue line denotes a single-plasmon picture, whilst the red line shows the results of a full quantum treatment, which is necessary for higher doping concentrations. The inset shows the absorption coefficient peak’s magnitude change as a function of the wide well doping.

Figure 2 shows that the calculated absorption peak for the moderate Ga-doping values ($N_{D}=3\times 10^{18}\;{cm}^{-3}$) was approximately 70 meV. The difference between the single- and multisubband-plasmon pictures was only a couple of meV. Comparatively, for the large values of Ga doping ($N_{D}=5\times 10^{19}\;{cm}^{-3}$), we noticed that in a single-plasmon picture, the absorption peak was approximately 100 meV, whilst in the multisubband-plasmon picture, the peak was around 170 meV, which represents a significant difference, showing that the effect of the depolarisation shift cannot be disregarded. The more we increased the doping, the more pronounced the depolarisation shift became. This is also illustrated in Figure 3, in which the energy corresponding to the absorption peak is plotted as a function of the doping density. The inset in Figure 3 shows the dependence of the absorption coefficient on the doping density.

**Figure 4:**
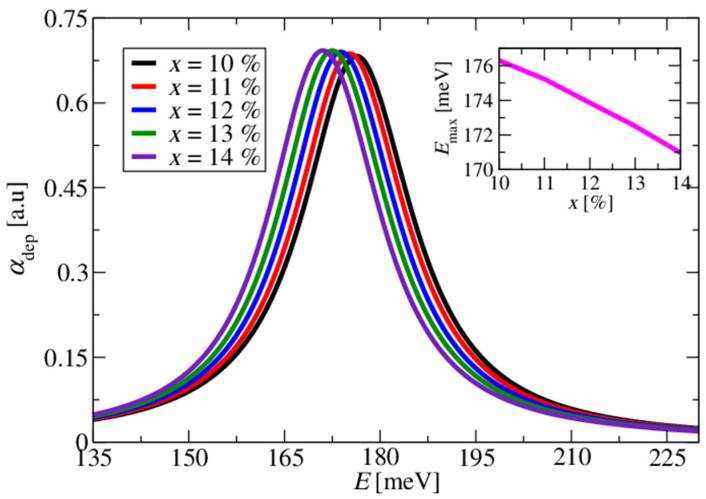
Absorption spectra for differing values of the Mg composition in the barrier layers. The doping density of the wide well was set to $5\times 10^{19}\;{cm}^{-3}$, and a lattice temperature of *T* = 300 K was used in all simulations. The inset shows the energy that corresponds to the absorption spectrum peak position as a function of the Mg composition in the barrier layers. As the percentage of Mg increased, the absorption spectrum peak redshifted by approximately 5 meV.

**Figure 5:**
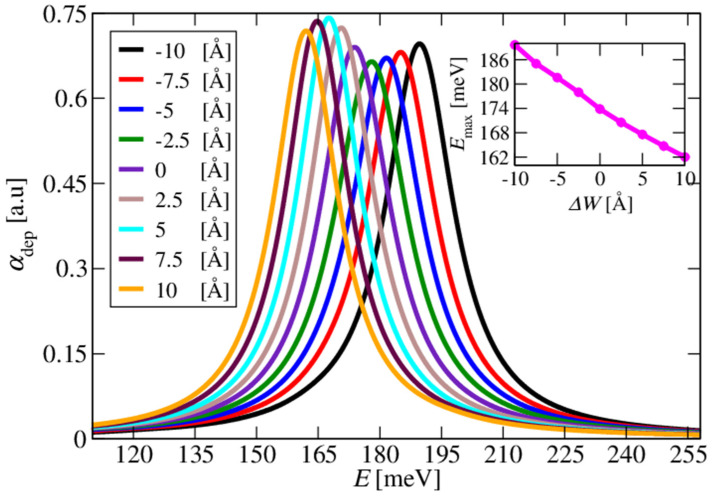
An expansion of the well width redshifted the absorption spectra. The inset shows the absorption peak energy as a function of the change in the well width, Δ*W*.

In Figure 4 and Figure 5, we show the calculated absorption for the large doping concentration of $N_{D}=5\times 10^{19}\;{cm}^{-3}$, accounting for the depolarisation field, i.e., in the full multisubband-plasmon picture. The change in the conduction band offset (CBO) by sweeping the percentage of Mg in the barrier layers from 10% to 14% with a 1% increment resulted in the shift in the absorption spectra, as shown in Figure 4. The absorption peak energy redshifted as the Mg percentage in the barrier increased. The impact of the slight variations in the width of the doped well by ±2.5Å steps on the absorption spectra is shown in Figure 5. Increasing the well width blueshifted the absorption spectra.

### 3.2. Resonant Tunnelling Structures

**Figure 6:**
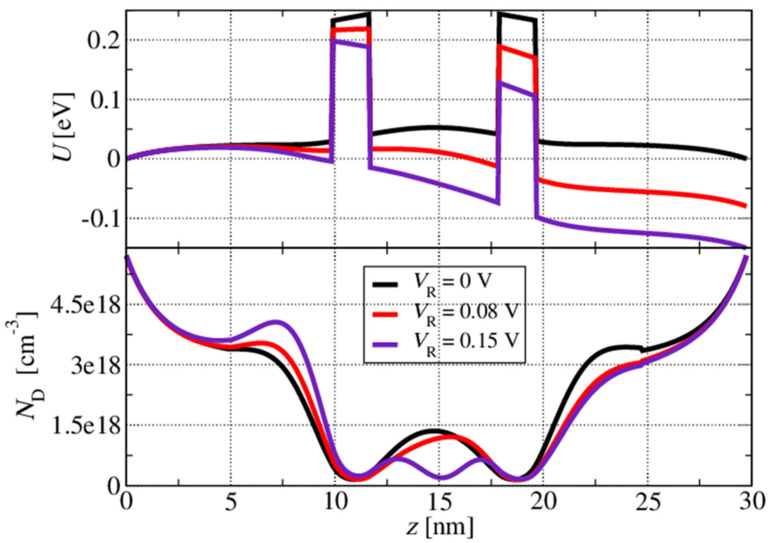
Self-consistent potential and corresponding electron concentration for three different biasing conditions.

In this subsection, we focus on the resonant tunnelling structures. In the first set of calculations, we analyse a nonpolar m-plane ZnO/ZnMgO double-barrier resonant tunnelling structure to investigate the effect of the layer thickness fluctuation and the impact of the doping density variation on the resonant tunnelling performance of the structure. The first analysed structure had a 6 nm thick ZnO quantum well surrounded by two Zn_88_Mg_12_O barriers, each 2 nm thick. The layer thicknesses and Mg composition in the barrier layers were chosen to mimic the resonant tunnelling (i.e., electron injection) part of the prospective quantum cascade structure. The double-barrier structure was placed between the injector and collector ZnO layers, and an external bias, $V_{R}$, in the range between 0 and 0.25 V was applied to this short structure. The Ga doping of the injector/collector was set to be $N_{D}=3\times 10^{18}\;{cm}^{-3}$. The self-consistent effective potential and corresponding electron concentration at the lattice temperature of *T* = 300 K for the three different biasing conditions are shown in Figure 6. It can be seen that the conduction band edge at the centre of the quantum well is bent upwards due to the increased electron population in the lowest quasi-bound level at lower voltages, as it is closer to the Fermi energy in the highly doped emitter side. Thus, the larger electron concentration in the quantum well led to a stronger self-consistent field, resulting in larger band bending. For the larger applied voltages (for example, $V_{R}=0.15\;V$ in Figure 6), the curvature of the self-consistent electron concentration in the well region changed its shape as the effective potential in the well region dropped, i.e., the second quasi-bound state in the well region accumulated electrons and became relevant for the carrier tunnelling process.

**Figure 7:**
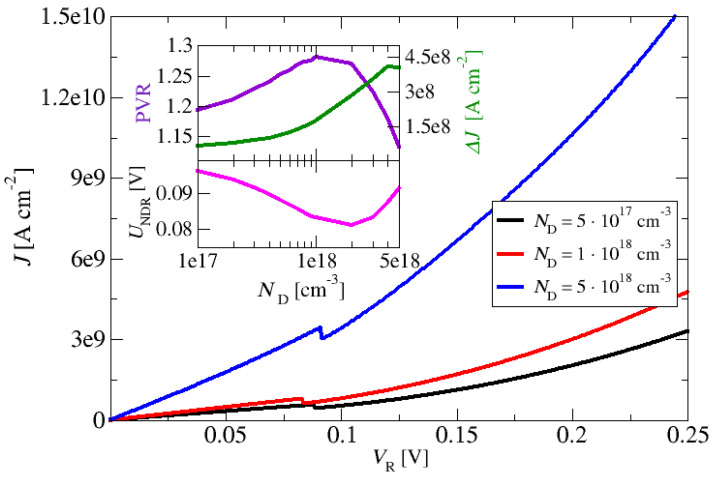
Current density–voltage characteristics of nonpolar m-plane ZnO/Zn_88_Mg_12_O resonant tunnelling structures. The doping level of the emitter and collector on the current density varied in the range from $10^{17}\;{cm}^{-3}$ to $5\times 10^{18}\;{cm}^{-3}$. The layer thickness barriers and quantum wells of the constituent epi-layers of the structure, starting from the emitter, in nm, are 10/**2**/6/**2**/10 (thicknesses of the quantum barriers are marked in bold). The inset shows the current-density peak-to-valley ratio (upper panel, left-hand *y*-axis) and current-density peak-to-valley difference (upper panel, right-hand *y*-axis) at the NDR; the NDR voltage as a function of the emitter is shown in the lower panel of the inset.

In the second set of calculations, the emitter and collector region doping of the m-plane ZnO-based double-barrier resonant tunnelling structure was varied from $\;10^{17}\;{cm}^{-3}$ to $5\times 10^{18}\;{cm}^{-3}$. For all simulations, the lattice temperature was again set to *T* = 300 K. Figure 7 shows the current density–voltage characteristics of the structure for three values of doping. The current density increased with the increase in the doping level of the emitter and collector over the whole range of bias voltages. Figure 7 also shows that for all investigated doping values, the current density–voltage curves had a region with negative differential resistance (NDR).

The peak-to-valley ratio is the ratio between the local maxima and the local minima around the NDR points in the RTD’s current–voltage (*I–V*) characteristic. Nominally, RTDs can have multiple resonances depending on the design of the electron subband states; in practical cases, the most important is the first “hump” in the *I–V*. Another important metric for RTDs is also the dynamic range that, mathematically, is the difference between these current values rather than their ratio. Depending on the application, the dynamic range may also be a very important figure of merit for an RTD’s performance. From the insets in Figure 7, one can see that the current density peak-to-valley ratio (indicating the quality of the peak separation); the peak-to-valley difference of the current density, ΔJ, at the NDR; and the voltage value, V_NDR_, in the NDR region depend on the doping level of the emitter and collector. The PVR increased with an increase in the doping level of the emitter and collector and had a maximum PVR of ~1.255 at the doping concentration of $10^{18}\;{cm}^{-3}$. Further increases in the doping level of the emitter and collector led to a decrease in the PVR, and at a doping concentration above $5\times 10^{18}\;{cm}^{-3}$, the region with the NDR feature almost disappeared (PVR~1). From these results, it can be anticipated that an optimal n-type doping level of the emitter and collector for this structure is approximately $10^{18}\;{cm}^{-3}$.

**Figure 8:**
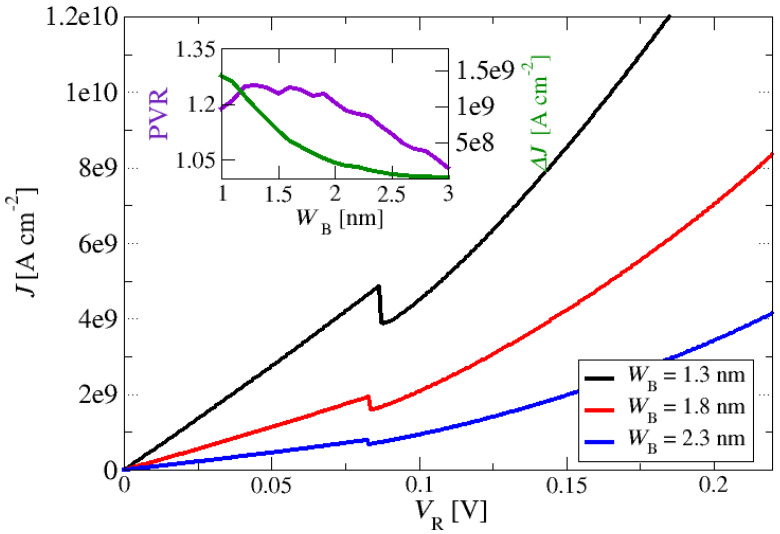
Current density–voltage characteristics of nonpolar m-plane ZnO/Zn_88_Mg_12_O double-barrier resonant tunnelling structures. The thicknesses of the barriers, *W*_B_, were exposed to monolayer-scale fluctuations at approximately a nominal value of 2 nm. The nominal layer thicknesses of the barriers and the quantum well of the constituent epi-layers of the structure, starting from the emitter, in nm, were 10/**1**–**3**/6/**1**–**3**/10 (thicknesses of the quantum barriers are marked in bold). The inset shows the current-density peak-to-valley (PVR) ratio (left-hand *y*-axis) and current-density peak-to-valley difference (right-hand *y*-axis) at the NDR; a doping-density value for the emitter/collector of $3\times 10^{18}\;{cm}^{-3}$ and a lattice temperature of *T* = 300 K were used in all simulations.

As pointed out earlier, the ZnO/ZnMgO material system has attracted much interest recently even though the crystal growth of this system is technologically challenging. Furthermore, layer thickness variation and interface roughness on the order of a fraction of a monolayer are other issues that arise [63]. Structures based on resonant tunnelling mechanisms like THz QCLs [46] require high-quality and very precise growth of the layer structures to provide efficient electron resonant tunnelling and a selective injection transport process into the upper laser level. To analyse the impact of the layer thickness fluctuation in the ZnO/Zn_88_Mg_12_O resonant tunnelling structure, we performed one more set of current density–voltage characteristics simulations in a reference double-barrier resonant tunnelling structure with a nominal barrier thickness of approximately 2 nm and a well thickness of 6 nm. This structure is similar and mimics the resonant injection layers in the THz quantum cascade structure in Ref. [46]. A doping-density value of $3\times 10^{18}\;{cm}^{-3}$ in the emitter and collector regions and a temperature of *T* = 300 K were used in the simulations. As shown in Figure 8, the monolayer fluctuation of the Zn_88_Mg_12_O barrier thickness, *W*_B_, despite its relatively low Mg composition, would have an important impact on the magnitude of the tunnelling current, PVR and current-density peak-to-valley difference, indicating that it is as an important parameter in prospective ZnO/ZnMgO THz QCL electron transport optimisations. The inset in Figure 8 shows that an optimal value of around 1.3 nm was predicted for this particular resonant tunnelling structure.

**Figure 9:**
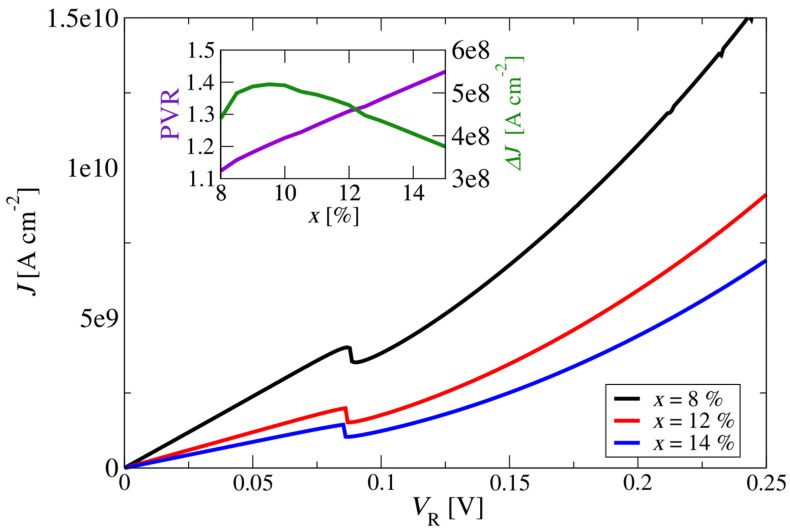
Current density–voltage characteristics of nonpolar *m*-plane ZnO/Zn_1−x_Mg_x_O double-barrier resonant tunnelling structures. An optimal doping density for the emitter/collector of 1 × $10^{18}\;{cm}^{-3}$ and a barrier thickness of 1.3 nm were chosen, i.e., the nominal layer thicknesses of the barriers and the quantum well of the constituent epi-layers of the structure, starting from the emitter, in nm, were 10/**1.3**/6/**1.3**/10 (thicknesses of the quantum barriers are marked in bold). The inset shows the current-density peak-to-valley (PVR) ratio (left-hand *y*-axis) and current-density peak-to-valley difference (right-hand *y*-axis) at the NDR; a lattice temperature of *T* = 300 K was used in all simulations.

Finally, to analyse the impact of the Mg mole fraction variation on an RTD with (previously obtained) an optimised doping density of *N_D_* = 1 × $10^{18}\;{cm}^{-3}$ and a barrier thickness of *W_B_* = 1.3 nm, we performed the final set of current density–voltage characteristics simulations. As shown in Figure 9, the Mg variation in the Zn_1−x_Mg_x_O barriers would, again, have a relevant impact on the magnitude of the tunnelling current, PVR and current-density peak-to-valley difference, indicating that it is an additional important parameter in prospective ZnO/ZnMgO THz QCL electron transport optimisations. The inset in Figure 9 shows that a value of approximately x = 9% would produce a maximised value of the current-density peak-to-valley difference, Δ*J*, in this particular structure.

## 4. Conclusions

Experimental realisations of high electron–LO-phonon resonance nonpolar m-plane ZnO/ZnMgO-based intersubband heterostructures demand comprehensive but still simple theoretical modelling and analysis of the coherent tunnelling transport and intersubband optical absorption. We modelled the absorption in highly doped MQW structures, which mimicked a QCL active region, and discussed the importance of a depolarisation shift in the ZnO/ZnMgO light-absorbing/emitting ISBT structures. Furthermore, we modelled the current–voltage characteristics and analysed the electron density distribution as a function of the voltage applied to the double-barrier resonant tunnelling structure. The calculations show that tunnelling current PVR is very sensitive both to small (monolayer) barrier thickness variations and to the percentage of Mg mole fraction changes, as well as to the injector/collector doping density. This information is useful for optimising resonant tunnelling electron transport and injection efficiency in perspective structures like nonpolar m-plane ZnO-based heterostructures operating in the THz frequency range.

## Data Availability

The data associated with this paper are openly available from the University of Leeds Data Repository: https://doi.org/10.5518/1490.

## Appendix A

$U_{xc}$ is the local exchange-correlation potential expressed as follows [64]:

(A1) $$U_{xc}\left(z\right)=-\frac{e^{4}}{32\pi^{2}\hbar^{2}}\frac{m^{*}\left(z\right)}{{\varepsilon^{*}\left(z\right)}^{2}}{\left(\frac{9\pi }{4}\right)}^{\frac{1}{3}}\frac{2}{\pi r_{s}^{*}}\left[1+0.054r_{s}^{*}log\left(1+\frac{11.4}{r_{s}^{*}}\right)\right],\;$$

where $\varepsilon^{*}\left(z\right)$ is the dielectric constant and $r_{s}^{*}$ is the average distance between carriers scaled by the effective Bohr radius:

(A2) $$r_{s}^{*}=\sqrt[{3}]{\frac{3}{4\pi n\left(z\right)}}\frac{1}{a_{B}^{*}},\;$$

(A3) $$a_{B}^{*}=\frac{4\pi \varepsilon^{*}\left(z\right)\hbar^{2}}{m^{*}\left(z\right)e^{2}},\;$$

## Appendix B

The Hamiltonian describing the intersubband plasmon can be written as follows [32]:

(A4) $$H_{plasmon}=\sum_{\alpha }\hbar {\tilde{\omega }}_{\alpha }p_{\alpha }^{†}p_{\alpha }+\frac{\hbar }{2}\sum_{\alpha \neq \beta }\Xi_{\alpha ,\beta }(p_{\alpha }+p_{\alpha }^{†})(p_{\beta }+p_{\beta }^{†}),\;$$

where $p_{\alpha }$ is the destruction operator of the intersubband plasmons α; ${\tilde{\omega }}_{\alpha }=\sqrt{\omega_{\alpha }^{2}+\omega_{P\alpha }^{2}}$ is the plasma-shifted transition frequency, where $\omega_{P\alpha }^{2}=\frac{2e^{2}\Delta N_{\alpha }\omega_{\alpha }}{\hbar \varepsilon_{0}\varepsilon_{s}}S_{\alpha \alpha }$. The coupling due to dipole–dipole Coulomb interactions is described by the coupling strength $\Xi_{\alpha ,\beta }$:

(A5) $$\Xi_{\alpha ,\beta }=\frac{\omega_{P\alpha }\omega_{P\beta }}{2\sqrt{{\tilde{\omega }}_{P\alpha }{\tilde{\omega }}_{P\beta }}}C_{\alpha \beta },\;$$

(A6) $$C_{\alpha ,\beta }=\frac{S_{\alpha \beta }}{\sqrt{S_{\alpha \alpha }S_{\beta \beta }}},$$

Where $S_{\alpha \beta }$ is the characteristic length that depends on the overlap between microcurrents, given as follows:

(A7) $$S_{\alpha \beta }=\frac{1}{\hbar \omega_{\alpha }}\frac{1}{\hbar \omega_{\beta }}{\left(\frac{\hbar^{2}}{2m^{*}}\right)}^{2}\int_{-\infty }^{+\infty }dz\xi_{\alpha }(z)\xi_{\beta }(z).\;$$

Diagonal term $S_{\alpha \alpha }$ refers to the interaction between dipoles associated with the same transition, whilst $S_{\alpha \beta }$ are the dipoles belonging to different transitions. In Equation(A7), the term $\xi_{\alpha }(z)$ is given by the following:

(A8) $$\xi_{\alpha }\left(z\right)\equiv \xi_{ij}\left(z\right)=\psi_{i}\left(z\right)\frac{\partial \psi_{j}\left(z\right)}{\partial z}-\psi_{j}\left(z\right)\frac{\partial \psi_{i}\left(z\right)}{\partial z}.\;$$

The coupling between *N* intersubband plasmons leads to the multisubband plasmons due to Coulomb interactions. The new *N* frequency, $W_{N}$*,* can be calculated by diagonalising the following 2N×2N matrix [32]:

(A9) $$M=\left[\begin{array}{cccc} I_{1} & C_{12} & \ldots & C_{1N}\\ C_{12} & I_{2} & \ldots & C_{2N}\\ \vdots & \vdots & \ddots & \vdots \\ C_{1N} & C_{2N} & \ldots & I_{N} \end{array}\right]\;$$

(A10) $$M=\left[\begin{array}{cc} \omega_{\alpha } & 0\\ 0 & -\omega_{\alpha } \end{array}\right],\;M=\left[\begin{array}{cc} \Xi_{\alpha ,\beta } & -\Xi_{\alpha ,\beta }\\ \Xi_{\alpha ,\beta } & -\Xi_{\alpha ,\beta } \end{array}\right].\;$$

Each new eigenmode of the system $W_{n}$ is associated with the excitation of a multisubband plasmon, described by the following operators:

(A11) $$P_{n}=\sum \left(a_{n\alpha }p_{\alpha }+b_{n\alpha }p_{\alpha }^{†}\right),\;$$

where components of the eigenvectors $V_{n}={\left(a_{n1},b_{n1},\ldots ,a_{nN},b_{nN}\right)}^{T}$ satisfy the bosonic normalisation condition:

(A12) $$\sum \left({\left|a_{n\alpha }\right|}^{2}-{\left|b_{n\alpha }\right|}^{2}\right)=1.\;$$

We can now write the Hamiltonian (A4) using the multisubband plasmons modes as follows:

(A13) $$H_{plasmon}=\sum_{n}\hbar W_{n}P_{n}^{†}P_{n}.\;$$

The current density for *n*-th multisubband plasmon is written as follows:

(A14) $$J_{n}(z)=\frac{e\hbar }{2m^{*}\sqrt{S}}W_{n}\sum_{\alpha }\frac{\xi_{\alpha }\left(z\right)\sqrt{∆N_{\alpha }}}{\sqrt{\omega_{\alpha }{\tilde{\omega }}_{\alpha }}}{\left(a_{n\alpha }+b_{n\alpha }\right)}^{-1}.\;$$
